# An Empirical Study on the Influence Path of Environmental Risk Perception on Behavioral Responses In China

**DOI:** 10.3390/ijerph16162856

**Published:** 2019-08-10

**Authors:** Shan Gao, Weimin Li, Shuang Ling, Xin Dou, Xiaozhou Liu

**Affiliations:** School of Public Administration, Central South University, Changsha 410083, China

**Keywords:** environmental risk, risk perception, behavioral responses, influence path

## Abstract

In recent years, the outbreak of numerous environmental risk incidents aroused widespread public concern about the amplification mechanism of environmental risk in China. However, few studies have investigated the influence path of environmental risk perception on behavioral responses in Chinese context from a micro perspective. In this article, we develop a multidimensional path model from environmental risk perception to behavioral responses, which aims to investigate how the public’s environmental risk perception influence its different behavioral responses, including environmental radical behavior, environmental concern behavior and environmental protection behavior. A survey data from Chinese General Social Survey 2013 (CGSS2013), was used to test the model, including questions related to information channel (e.g., media use, interpersonal network), cognitive evaluation (e.g., environmental value, public’s evaluation of environmental governance) and environmental knowledge. The results indicate that both information channel factors and cognitive evaluation factors can significantly influence the progress that from environmental risk perception to behavioral responses, of which both media use and environmental value play the amplification role in the transform process, while interpersonal network and public’s evaluation of environmental governance would inhibit public’s risk perception transforming into environmental behavior. Besides, environmental knowledge displays a key bridge role between information channel factors and cognitive evaluation factors. This research findings also demonstrated the evolution paths of three environmental behaviors under the same level of risk perception, namely, risk transformation and diffusion, risk perception enhancement and risk attenuation respectively.

## 1. Introduction

In recent years, China has witnessed numerous large-scale street protests and petitions against the construction of major projects such as chemical plants, waste disposal facilities, airports, nuclear power plants and freeways due to the potential environmental damage that may be caused by the above-mentioned facilities [1]. For instance, over 3000 local citizens filled Nanping Square on May 2013 to protest the construction of a *para*-xylene (PX) chemical plant in Kunming, even though experts claimed that the risk from this project is rather low. Similar environmental protests have also ocurred in many other Chinese cities such as Xiamen, Beijing, Ningbo, Guangzhou, Dalian, Wuxi, etc. [2]. These environmental protests have aroused widespread public concern about the amplification mechanism of environmental risk in China. That is, why a small risk assessed by risk experts can attract wide public attention, and then become intertwined with economic interests and public protection of rights, which has a profound impact on society and even moves towards group struggle. 

To better understand the phenomenon of environmental risk amplification in China, previous literatures have applied the social amplification of risk framework (SARF) in various contexts, such as food safety [3,4], public health emergencies [5,6,7] and natural hazards [8,9,10], to understand individual responses as well as information transfer related to a variety of risks and risk-related events. In terms of the stage of information transmission, most studies focused on investigating the role of social amplification station in the progress of risk amplification. For example, authors such as McComas demonstrated that attending a public meeting can act as a “social amplification”, but the role on public’s risk perception largely relied on the interaction among the public, officials and other risk sources [11]. Besides, other authors such as Chung have indicated that the internet can provide more efficient information channels for interactive communication and an open space for active information sharing and public participation compared with traditional media [12]. Furthermore, Chong and Choy take a similar view, that is, they found Facebook to have an amplifying effect on emotions that the other media (e.g., mainstream newspapers, online forums) didn’t have [6]. In terms of the stage of response, most authors have paid more attention to the ripple effects. For example, Ichinosawa indicated the post-tsunami tourism decline is a complex process involving risk-induced stigmatization of the region and historically embedded vulnerabilities in the local society [13]. However, despite the fact SARF provides more specific details about individual, organizational, and social coping actions triggered by risk events, little research has provided any empirical explanation about the internal mechanism whereby the public’s environmental risk perception influences it’s behavioral responses in a Chinese context. Besides, this framework is also not explained well regarding what kind of specific actions an individual may respond with when a specific scenario is involved. Therefore, it is crucial to investigate how the public perceives environmental risk about environmental issues, and how the public’s environmental risk perception influences their subsequent behavioral intentions. To address these issues, we constructed a hypothetical model to understand that how public’s environmental risk perception is transformed into different behavioral responses in China, such as radical environmental behavior, concerned environmental behavior and environmental protection behavior.

Previous studies have employed structural equation models (SEM) to investigate which factors influence the public’s risk perception and it’s behavioral responses. Of these, some authors such as Kuttschreuter [14], Prati [15] or Allum [16] focus on examining the factors affecting the public’s risk perception. For example, Whitfield et al. explored how durable cognitive and cultural foundations, such as values, beliefs, and social expectations effect citizens’ risk perception of nuclear power by using the structural equation modeling method [17]. Allum employed the structural equation model to examine the relationship between trust and risk perceptions of genetically modified food [16]. In addition, other authors like Kuttschreuter [14] or Niles [18] have focused on paying attention to behavioral responses. That is, they mainly investigate how the public responds to risk, and suggest that understanding these risk responses is as important as understanding risk perception. For example, Kuttschreuter [14] developed a structural equation model to examine the psychological determinants of the responses to food risk messages. However, to date few studies have investigated how the public’s environmental risk perception influence its behavioral responses by using structural equation models. Therefore, following this stream of literature, this article develops a hypothesized framework to determine the factors that have an impact on responses to the environmental risk, based on a structural equation model.

The remainder of this article is organized as follows: First we propose a theoretical framework of how the public’s environmental risk perception influences its behavioral responses in Section 2. Section 3 details the methodology used in this study, while the main results are reported in Section 4. In Section 5, we provide a detailed discussion about the influence path of environmental risk perception on behavioral responses. Finally, some limitations of this study and future research directions, followed by the conclusions, are discussed in Section 6.

## 2. Theoretical Framework

**Figure 1:**
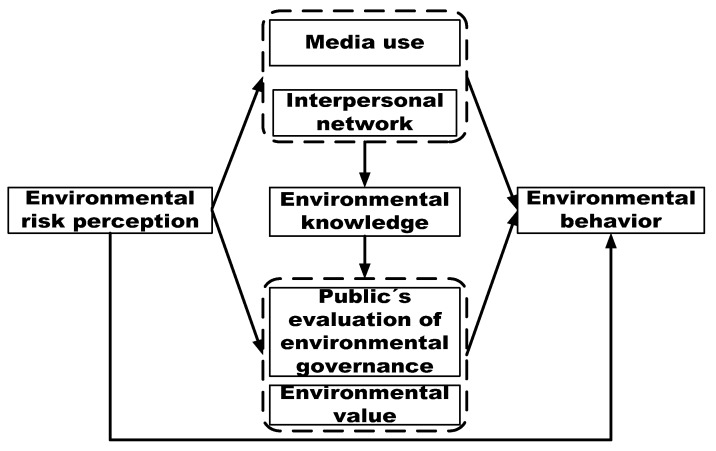
Theoretical framework for factors influencing environmental risk perception on its behavioral responses.

In the shift from risk perception to environmental behavior, complicated factors come into play simultaneously. In this article, we are interested in investigating the influence path of environmental risk perception on behavioral responses in the Chinese context. Based on the social amplification framework of risk and environmental practice, we develop a conceptual model to rethink that how public’s environmental risk perception is transformed into different behavioral responses in the Chinese context, which is depicted in Figure 1. Environmental behavior is the dependent variable that needs to be explained and environmental risk perception is the independent variable. Besides, the information channel aspect factors include media use and interpersonal networks, the cognitive evaluation aspect factors include environmental values and the public’s evaluation of environmental governance, and environmental knowledge is the intermediary factor that acts as the bridge between information channel aspect factors and cognitive evaluation aspect factors.

### 2.1. Environmental Risk Perception and Environmental Behavior 

Risk perception is a core factor influencing individuals’ responses to risk events. The so-called environmental risk perception denotes the intuitive judgment of individuals or social groups about environmental issues characterized with high complexity and uncertainty [19]. In general, risk characteristics (e.g., occurrence probability, degree of injury, uncertainty, and continuum of the aftermath), discrepancy of individual characteristics, and social psychological factors (e.g., trust, justice perception, political view, value, and identity) are the three main dimensions that affect public’s risk perception [20,21,22]. Environmental behavior can be classified into different types on the basis of different classification standards. For example, Rubin et al. indicated that conflictive actions can be divided into evasion, contention, concession, and problem-solving [23]. Besides, according to the level of aggressiveness, Caiani et al. showed that the public’s protest actions can be classified into conventional actions, demonstrative actions, expressive actions, confrontational actions and violent actions [24]. However, the existing studies on public attitudes and actions towards project site selections with existing environmental risk usually make a simple binary partition and mostly focus on public confrontational actions, whereas, no attention is paid to the complexity and dynamic convertibility of public’s risk coping actions. As authors like Lindell and Perry have shown, the public often displays three types of risk coping action: information searching action, problem-protection action, and emotion-focused action, on the basis of the PADM model [8], but this model has certain limitations in explaining the public choices of risk coping action, because it fails to present a specific behavioral image. Therefore, this article considers the level of risk perception and environmental practice in China, and divides environmental behavior into radical environmental behavior, environmental concern behavior and environmental protection behavior, of which, radical environmental behavior refers to environmental protests induced by existing environmental pollution or potential damage, which can produce a remarkable effect on social order. For example, in the 2007 “Anti-PX-Project” protest that took place in Xiamen city, citizens believed the plant would cause huge damage to their health, and living conditions and were motivated by this psychologically perceived risk to organize protests. Environmental protection behavior is a classic case of “negative aggressive” behavior that the public chooses because they are powerless to prevent environmental risk. In general, escape behavior (e.g., reducing the frequency of going out, never going to damaged areas) and security precaution actions (e.g., periodic physical examinations, stockpiling some materials that prevent environmental risk) are the two common environmental protection behaviors. Besides, environmental concern behavior refers to the public often actively discussing environmental issues with their relatives and friends or taking the initiative to focus on environmental issue that television and radio have reported. 

The relationship between risk perception and behavioral response to some specific risks has been extensively studied in the literature [25,26]. Previous studies have indicated that risk perception has a significant positive association with behavioral intention to participate in environmental action [27,28]. For example, Feng et al. indicated that the customer often selects to not to buy the original products, or choose to buy a high-quality product when facing product quality risks [29]. Kuttschreuter also takes a similar view, and has shown that individuals with a level of risk perception about food quality are more likely to take actions to avoid the risk [14]. Besides, Glaser indicated that individuals with a higher level of environmental risk perception would adopt radical risk coping actions, such as petitions, street protests, and violent incidents, whereas it is possible for the public to ignore a potential risk when they perceive a low environmental risk [30]. Therefore, with respect to environmental risk in China, we also want to examine whether individuals’ initial environmental risk perception has a positive influence on their behavioral intentions. 

### 2.2. Intermediary Factors: from Environmental Risk Perception to Its Behavioral Response

#### 2.2.1. Information Channel Factors

Many studies have explored the relationship between mass media and the social amplification of environmental risk [31,32,33]. Mass media has acted as one of the important predictors of environmental risk amplification. For example, Kasperson et al. indicated that mass media, as a “social amplification station”, can amplify or attenuate risk [32]. Renn also takes similar views, and has shown that mass media can interpret and represent signals, or risk information, to wider audiences [33]. Besides, Burns et al. also suggested that the behavior of mass media and the public play crucial roles in determining the impact of a hazardous event [34]. In general, environmental risk information disseminated through the media evoke public memories of similar experiences and even have an impact on other members of the public and groups who do not have direct experiences [35], promoting the occurrence of environmental behaviors. Hence, media use likely has an amplification effect on the public’s environmental risk perception and its behavioral response.

By contrast, interpersonal networks are also considered influential amplification stations. Previous papers have indicted that public experience and cognition of environmental risk are significantly related to interpersonal communication channels [36]. Besides, interpersonal networks have been shown to impact understanding and perception risks [37]. This is likely because interpersonal networks can provides an experience channel for public environmental risks, namely, the public can quickly recognize environmental risk information and shape environmental coping behaviors through their interpersonal networks. Thus, we hypothesize that interpersonal networks can likely influence the path that from environmental risk perception to its behavioral responses.

#### 2.2.2. Cognitive Evaluation Factors

Environmental value is another factor that may affect the path from environmental risk perception to its behavioral responses. Previous studies have indicated that the amplification of environmental risk is the result of interaction among specific value systems [38], especially, the change of public social values from materialism, which pays attention to material and safety needs, to post-materialism which emphasizes the belongingness, self-expression and quality of life, that may lead to the enhancement of public experience of environmental risks [39]. For example, Slimak and Dietz explored the relationship between environmental values and environmental risk perception, and showed that the NEP environmental values are important predictive factors of environmental risk perception [40]. In addition, Willis and DeKay indicated that individuals who agree with the NEP value may have a lower level or risk acceptance [41]. Therefore, we assumed that environmental value can play a significantly intermediary role in the progress that from environmental risk perception to environmental behavior.

Previous literature indicated that public trust in governmental agencies is an key factor that affects environmental risk [42]. An online investigation carried out in China found that 80% of the public in urban areas held distrustful attitudes toward government [43]. This means that public distrust of government would greatly influence the performance of environmental management in China [44]. Specifically, this distrust of government, on the one hand, may increases the public’s suspicion of the government’s ability to address environmental issues, and on the other hand, it also causes public suspicion of the environmental information communicated to the public [45,46], ultimately leading to public resistance to environmental risks. Terwel et al. also demonstrated that the extent of public trust in government affects the public’s inclination to protest [47]. That is, the higher the evaluation of governmental departments, the public’s behavioral responses may tends to be more stable [48]. Hence, we hypothesize that public’s evaluation of environmental governance likely influences the progress from environmental risk perception to environmental behavior.

#### 2.2.3. Bridge Factor

Environmental knowledge is an important factor that is hard to ignore when it comes to environmental risk perception [49]. In fact, individuals’ risk perception usually comes from their indirect experience, which means that they often assess the potential damage of environmental pollution issues by means of their own environmental knowledge [50]. In this sense, environmental knowledge can act as a key factor influencing the progress from risk perception to environmental behavior. On the one hand, information channels have a significant effect on the public’s environmental knowledge. In fact, individuals can acquire environmental knowledge through the following three channels: interpersonal networks, environmental education and media use. For example, according to a study related to social surveys from the USA, the public tends to use media, such as newspapers and the internet, to search for information related to climate change, which, to some extent, reflects the public’s knowledge acquisition behavior [51]. Besides, Karahan and Roehrig also indicated that social media can help individuals acquire more environmental knowledge [52]. On the other hand, individuals’ knowledge about environmental issues may be not correct [53], but the existing literature has demonstrated that the level of environmental knowledge that individuals perceive can also act as an important predictive factor of environmental protection behavior, which, to some extent, reflects the relationship between environmental knowledge and environmental behavior. Thus, we hypothesize that environmental knowledge acts as bridge factor between information channel factors and cognitive evaluation factors.

## 3. Methodology

### 3.1. Sample and Data Collection

The data used in this study are from the environmental module of China General Social Survey (CGSS2013) in 2013. The questionnaire survey adopts a multi-stage and hierarchical sampling design, and the participants are urban and rural residents over the age of 18 in China, covering 31 provinces in China. After the samples with serious lack of information are eliminated, we obtain 6135 valid samples. Specifically, the female samples entering the final analysis account for 49.70% while the male samples account for 54.10%; The samples aged from 18 to 60 account for 80.90% while the samples aged 60 and above account for 29.10%; The samples of party members account for 13.50% while the samples of non-party members account for 86.50%; The samples with no education background, primary or private school, junior middle school, high school (or technical secondary school, higher vocational school), junior college, undergraduate, graduate degrees and above account for 6.00%, 17.00%, 30.50%, 23.40%, 11.30%, 10.80% and 1.00% respectively; the rural samples account for 31.30%, while the urban samples account for 68.70%.

### 3.2. Measurement of Variables 

In this section, we presents the operationalized measures of all variables, including media use, interpersonal network, environmental knowledge, environmental value and the public’s evaluation of environmental governance.

*Environmental risk perception* (ERP) is an important construct to examine how individuals understand environmental risks, which mainly refers to the degree of environmental risk that the public perceives. Thus, in this study, we measured risk perception using two items, of which the first item is “do you know the following environment issues, such as air pollution, industrial wastes pollution, water pollution, food contamination, noise pollution, and solid waste pollution?”, and the second item is “If so, how severe are those environment pollution issues in your area?”. ERP is classified into seven levels (1 = no such problem, 2 = It doesn’t matter / hard to explain, 3 = general, 4 = not serious, 5 = not too serious, 6 = serious, 7 = very serious). The scores of each item of the scale are accumulated to obtain the continuous variable of environmental risk perception, and the higher the score is, the stronger the public perception of environmental risk is.

Facing environmental risk, individuals usually need more risk information about the environmental pollution issue to help assess environmental risk. Thus, information channel factors, including media use and interpersonal networks, are a key construct to investigate how individuals perceive and assess environmental risk. Of these, *media use* (MU) mainly refers to the frequency with which the participant uses media when facing potential environmental damage. MU is a continuous variable, and it is synthetically measured by a scale composed of six items (e.g., newspapers, magazines, broadcasts, internet and cellphones) in the CGSS2013 questionnaire, where the TV item is eliminated after the factor rotates. The answer assignment is classified into five levels (1 = never, 2 = very little, 3 = sometimes, 4 = often, 5 = very frequent). This mean that the higher the score is, the more frequently the public uses media.

Besides, the term *interpersonal network* (IN) refers to the frequency with which the participant contacts his /her friends. In this article, we measured interpersonal network through the following two aspects in CGSS2013 questionnaire: the frequency of social activities (e.g., watching TV, eating and play cards) with his/her friends or neighbors, and the frequency of playing a visit to his/ her relatives and friends. Obviously, IN is also continuous variable. The answer assignment is classified into five levels (1 = never, 2 = once or more times a year, 3 = once or more times a month, 4 = one to two times a week, 5 = almost every day). The measurement of IN can be obtain by accumulating the scores of related items of the scale, and the higher the score is, the stronger the public interpersonal relationship is.

*Environmental knowledge* (EK) is also another important factor that can influence the progress that from risk perception to environmental behavior, which mainly refers to individuals’ ability to recognize the sign, conceptions and behavior patterns related to environmental protection, and its measurement is mainly based on an environmental knowledge scale composed of ten items (e.g., automobile exhaust will not cause harm to human health; the excessive use of fertilizer and pesticide will exert a threat on environment; the formation of acid rain is unrelated to coal firing, etc), which has prove that the scale has good reliability and validity in practice in plenty of studies by Hong [54], but it needs to be noted that the items of 1, 3, 5, 7 and 9 are reverse items, and thus we must compute the EK score of each participant. EK is a binary variable, coded 1 if each actual judgment for the participant about environmental problem is correct; it is coded 0 if the participant doesn’t know or makes a mistake in responding to environmental questions.

Previous literatures have indicated that cognitive evaluation factors, including environmental value and the public’s evaluation of environmental governance, can significantly influence public’s environmental behavior. Of these *environmental value* (EV) refers to the value that deals with the relationship between the environment and people. EV is a sequence variable, and the answer assignment is classified into three levels (1 = environmental problem as the third social problem to be solved. 2 = the environmental problem as the second social problem to be solved, 3 = the environmental problem as the first social problem to be solved). That is, the higher the degree of public recognition, the stronger the demand for environmental improvement, and the more correct the environmental value. 

The public’s *evaluation of environmental governance* (EEG) refers to the satisfaction level of the public with the government’s environmental governance, which is measured through the following two aspects: the performance of environmental protection of the central government, and the performance of local governments in the most recent five years. According to Shi et al., there is a significant positive correlation between the public’s evaluation of environmental governance at a local level and environmental behavior, and a significant negative correlation between the public’s evaluation of environmental governance at a central level and environmental behavior [55]. Thus, in this article, we represent the overall score of evaluation of environmental governance by an equation that adds the score of the public’s evaluation of environmental governance at a central level and the score in the local level together. The answer assignment is classified into five levels (1 = one-sided emphasis on economic development and neglect of environmental protection, 2 = insufficient attention to environmental protection and insufficient investment in environmental protection, 3 = Although efforts have been spared, the effect is not good, 4 = Great efforts have been spared, and certain results have been achieved, 5 = Great achievements have been made), that is, the higher the degree of recognition is, the higher the evaluation of environmental governance is.

To measure the public’s behavioral responses when facing environmental risk, ten items in the CGSS2013 questionnaire were adapted, including environmental protests, actively focusing on environmental issues and related information through radio and television, discussing environmental pollution issues with relatives and friends, putting garbage into different trashcans, going shopping with his/her shopping basket, repeatedly using packaging bags, donations for environmental protection, actively participating in environmental protection activities organized by government departments or environmental NGOs, and planting forests or green areas at his/her own expense. The answer assignment is classified into three levels (1 = never, 2 = occasionally, 3 = often). In this article, we would obtain different types of environmental behaviors through accumulating the scores of related items of the scale, and the higher the score is, the more the public engages in environmental behaviors. We call it as environmental protection behavior (EPB), environmental concern behavior (ECB) and environmental radical behavior (ERB), respectively.

### 3.3. Descriptive Statistics and Correlations

Table 1 presents the descriptive statistics and bivariate correlations of all the constructs in this model. 

The participants reported average levels of environmental risk perception (M = 26.84), which is below the median, that is 28.13. It means that most of survey respondents have a relatively low environmental risk perception. Besides, the average level of both media use (M = 10.66) and interpersonal network (M = 8.12) are higher than their median, namely, 10.446, 8.04 respectively, which indicates that most of participants have high usage frequency of mass media, and strong social ties. Similarly, the sample distribution of both environmental value and public’s evaluation of environmental governance is the same as the information channel factors, that is, the survey respondents reported relatively high levels of environmental value (M = 0.32) and the public’s evaluation of environmental governance (M = 6.15), which are above the median, that is, 0.20 and 6.31, respectively. In terms of environmental knowledge, it should be noted that the average level of environmental knowledge (M = 5.71) is lower than its median, namely, 5.86, which means that most of participants have a low knowledge level. Besides, for those three type of environmental behavior, such as radical environmental behavior (M = 1.13), environmental protection behavior (M = 11.35) and environmental concern behavior (M = 3.52), all of them are higher than their median, namely, 1.12, 11.1231, 3.49, respectively. With respect to the correlations between various variables, there were significant high associations between many variables and no association between a few others. For example, environmental risk perception can significantly affect environmental behavior, and produces significant indirect effects through other variables. Given that there were sufficiently high inter-correlations for several pairs of the constructs, it is appropriate to discuss the influence of risk perception and intermediary variable, including interpersonal network, media use, environmental value, environmental governance evaluation and environmental knowledge, on environmental behavior, which is presented in the next section.

## 4. Results

In the shift from risk perception to environmental behavior, complicated factors come into play simultaneously, which need to be explained by multivariate methods. As is well-known, structural equation modeling (SEM), one of the most popular multivariate analysis tools, is often used to evaluate complex models in which intermediary effects are significant [56]. Therefore, in this article, we decided to test our postulated model through the structural equation modeling procedure using the AMOS software. 

### 4.1. Reliability and Validity Assessment

To verify the the hypothesized relationships of the conceptual model in Figure 1, we conducted a confirmatory factor analysis (CFA) using the AMOS software. Table 2 shows an acceptable model fit for three model, of which, model 1 represents the influencing path from risk perception to environmental radical behavior, model 2 represents the influencing path from risk perception to environmental concern behavior, and model 3 represents the influencing path from risk perception to environmental protection behavior. Specifically, in those models, we find that the normed chi-square ratio in model 1, 2, and 3 are 14.053, 40.076 and 24.869, respectively, which are above the desired cutoff value of 3.0, although all models achieved statistical significance at the level of 0.05. One important reason might be that the chi-square value of model is vulnerable to sample size. That is, the greater the sample size, the larger the chi-square value of model. Thus, we would adopt other indicators, such as NFI, CFI and RMSEA, to estimate the fit degree of model. Specifically, all root mean square errors of approximation (RMSEAs) in the three models are 0.046, 0.080 and 0.062, respectively, which are lower than 0.08, indicating a good fit. Besides, all the normed fit indexes (NFI = 0.954, 0.902 and 0.937) and comparative fit indexes (CFI = 0.957, 0.904 and 0.939) in the three models are both greater than 0.90. To summarize, the results suggest that the structural models have a good fit.

### 4.2. The Dynamic Evolution Progress of Environmental Behavior

**Figure 2:**
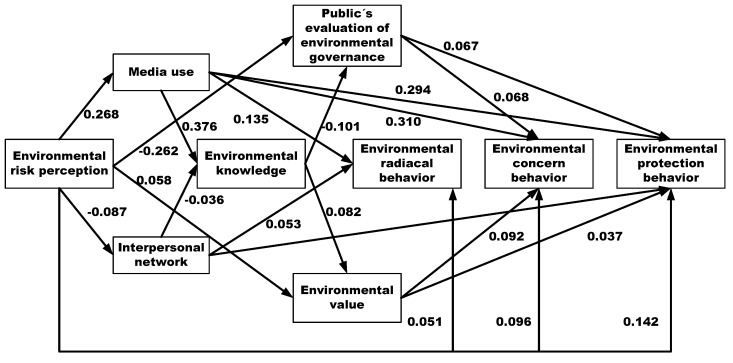
The dynamic evolution progress of environmental behavior.

As shown in the results of the structural equation modeling procedure in Figure 2, we can find that the overall performance of the model can effectively explain the dynamic evolution progress of environmental behavior, because all but three paths achieved statistical significance at the level of 0.05 or better and the performance of most significant path coefficients was also as expected. Specifically, both environmental value (*p* = 0.375 > 0.05) and public’s evaluation of environmental governance (*p* = 0.128 > 0.05) fail to predict environmental radical behavior, and interpersonal network (*p* = 0.077 > 0.05) also fails to directly predict environmental concern behavior. 

In this model, five mediated variables, such as media use, interpersonal network, environmental knowledge, environmental value and public’s evaluation of environmental governance were selected for the multivariate path analysis, and the relationships between variables were calculated, which is depicted in Figure 2. Specifically, the overall model shows that environmental risk perception is an antecedent variable, and it has the direct and indirect effect on different type of environmental behavior. For the direct effect, individuals who have higher level of environmental risk perception usually are more likely to take the corresponding action, such as environmental radical behavior (β = 0.089, *t* = 6.598), environmental protection behavior (β = 0.142, *t* = 11.031) and environmental concern behavior (β = 0.096, *t* = 7.514), to response environmental risk. 

Moreover, we found that public’s risk perception can also indirectly indicate its behavior responses. It means that risk perception is not the only factor of environmental behavior, and many other mediated variables, such as media use, interpersonal networks, environmental knowledge, environmental values and the public’s evaluation of environmental governance, also play an important role in the progress from risk perception to environmental behavior. For example, environmental risk perception was found to positively and significantly influence media use (β = 0.268, *t* = 21.841) and environmental value (β = 0.058, *t* = 4.544), whereas it may produce the negative effect on interpersonal network (β = −0.087, *t* = −6.822) and public’s evaluation of environmental governance (β = −0.262, *t* = −21.378). 

For information channel factors, the overall model in Figure 2 shows that both media use and interpersonal network are related to environmental behavior. For example, in the evolution progress of environmental radical behavior, both media use and interpersonal network can increase the probability of radical environmental behavior, that is, individuals with higher usage frequency of mass media (β = 0.135, *t* = 10.366), or with stronger social ties (β= 0.053, *t* = 4.232) would be more likely choose radical behavior to respond to environmental risks. For environmental protection behavior, we find that both media use and interpersonal networks, as the important parts of the information transmission progress, also directly influence environmental protection behavior. Namely, higher usage frequency of mass media can lead to a willingness to adopt environmental protection behavior in response to environmental risk (β = 0.294, *t* = 23.741). Similarly, stronger social ties can also positively and significantly influence environmental protection behavior (β = 0.051, *t* = 4.256). However, in the evolution progress of environmental concern behavior, the results in Figure 2 indicated that interpersonal networks fail to directly predict environmental concern behavior, because the coefficient of the path from interpersonal networks to environmental concern behavior fails to reach statistical significance at the level of 0.05 (*p* = 0.077 > 0.05), while media use was found to positively and significantly influence public’s environmental concern behavior. That is, individuals with higher usage frequency of mass media may more likely adopt environmental concern behavior (β = 0.310, *t* = 25.085). 

Cognitive evaluation factors are the another type of factor that can lead to a willingness to take the corresponding action in response to environmental risk. In terms of environmental protection behavior, the results in Figure 2 indicated that both environmental values (β = 0.037, *t* = 3.120) and the public’s evaluation of environmental governance (β = 0.067, *t* = 5.377) are also positively related to environmental protection behavior. Similarly, in the evolution progress of environmental concern behavior, both environmental values and the public’s evaluation of environmental governance also positively and significantly influence individuals’ concern behavior intentions, that is, individuals with a stronger desire to protect the environment would be more likely select the environmental concern behavior (β = 0.092, *t* = 7.719). We also find that a higher level of satisfaction with the government’s environmental management can increases the tendency to select environmental concern behavior (β = 0.068, *t* = 5.489). However, in the evolution progress of environmental radical behavior, it should be noted that both the public’s evaluation of environmental governance (*p* = 0.128 > 0.05) and environmental value (*p* = 0.375 > 0.05) fail to predict environmental radical behavior.

In addition, it should be noted that environmental knowledge is is a key factor that acts as the bridge between media use, interpersonal network, environmental value and the public’s evaluation of environmental governance. That is, higher usage frequency of mass media can increases individuals’ environmental knowledge (β = 0.376, *t* = 31.850), while stronger social ties fail to bring more environmental knowledge (β = −0.036, *t* = −3.088). Individuals who have greater environmental knowledge would have a stronger desire to protect the environment (β = 0.082, *t* = 6.425), and a lower level of satisfaction with the government’s environmental governance performance (β = −0.101, *t* = −8.236). 

## 5. Discussion 

This study empirically tests the influencing path from environmental risk perception to its behavioral responses in China. In this section, we will discuss the main results, and then provide explanations and some additional clarifications about these results.

### 5.1. Influence Factors: from Environmental Risk Perception to Behavioral Response

By analyzing how individuals’ risk perception influences their different environmental behaviors in China, our study shows that environmental risk perception plays an important role in predicting individuals’ behavioral responses to environmental risk in China. That is, environmental risk perception is positively and significantly related to individuals’ behavioral responses, including radical environmental behavior, environmental concern behavior and environmental protection behavior. This finding, on the whole, is in accordance with those from most existing studies. For example, some authors as Lubell [18], or Park and Yang [28] indicated that environmental risk perception has a significant and positive association with behavioral intention to participate in environmental action, but the above-mentioned studies failed to distinguish the effect of risk perception on various behaviors. In fact, the existing literatures have worked to find the relationship between risk perception and various environmental behaviors. For instance, Wang et al. indicated that individuals with low risk perception often do not adopt effective self-protection actions, but they may adopt a radical environmental behavior, and even participate in social protests in serious cases once they perceive a high environmental risk [56]. Besides, Prati et al. also take a similar view, namely, they have shown that people who have higher levels of risk perception of pandemic influenza H1N1 are more likely to adopt health-related recommendations to avoid the risk [15]. However, in our article, we find that individuals with higher risk perception would be more likely adopt environmental behavior, regardless of which type of behavioral responses. 

Furthermore, this study also finds that complicated factors come into play simultaneously in the shift from risk perception to environmental behavior, that is, both information channel factors (such as media use and interpersonal networks) and cognitive evaluation factors (such as environmental values and the public’s evaluation of environmental governance) have significantly mediating effects in the progress from risk perception to environmental behavior. For media use, it is easy to understand its role as an “amplification station” in the progress from environmental risk to environmental behavior. On the one hand, individuals with higher risk perception of a certain environmental issue often have a high usage frequency of mass media. One important reason might be that, individually perceived risk depended in large part on personal experiences and wisdom in past decades, but the ability to obtain and process risk information was limited, which often make them have weaken, or even ignore environmental risk, but with the rapid development of information and communication technology, as well as the improvement of environmental protection awareness, citizens can easily search for risk information about environmental issue through mass media, such as broadcasts, newspapers, and social media, etc. This means that environmental risk information disseminated through mass media evokes public memories of similar experiences and even have an impact on other members of the public and groups who do not have direct experiences [35], promoting the occurrence of environmental behaviors. In fact, this result was largely consistent with findings from previous literatures in other risk situations. For example, Yang et al. have indicated that individuals’ risk perception can influence their judgement about risk information needs [57]. That is, individuals often tend to obtain risk information about food safety through various media when they have the high level of risk perception. 

As for interpersonal networks, in this article, it should be noted that interpersonal networks had an inhibitory effect in the progress from risk perception to environmental behavior. On the one hand, most authors, such as Kasperson, have shown interpersonal networks can strengthen individuals’ risk perception [31]. Besides, Lindell and Hwang also take a similar view, and have shown that public experience and cognition of environmental risks are significantly related to interpersonal communication channels [36]. This means that interpersonal networks can provide an experience channel for individuals to quickly recognize environmental risk information and shape their environmental coping behaviors. However, in our study, we find that risk perception is negatively related to interpersonal networks, which means that a higher level of risk perception can decrease the frequency of social contacts. One important reason might be that a strong interpersonal network can help individuals obtain reliable risk information by information communication, as well as exchange their risk perception on a certain environmental issue, and then decrease the uncertainty of risk [58]. On the other hand, our results have indicated that individuals with stronger social ties would be willing to take corresponding environmental action, which is consistent with the existing studies. In summary, our results have confirmed that interpersonal networks can inhibit the public’s risk perception transformation into environmental behavior by using empirical research.

Meanwhile, this article also shown that cognitive evaluation factors, as another important mediated factor, would significantly influence the progress from risk perception to environmental behavior. For environmental values, it can perform as an “amplification station” in the progress from risk perception to environmental behavior. On the one hand, we find that individuals with a higher level of risk perception would tend to report their desires to improve the environment. That is, individuals may have a strong desire to take corresponding action to protect the environment, once they perceive potential damage from an environmental issue, which was demonstrated in the study of Gong [59]. On the other hand, for the relationship between environmental value and environmental behavior, only a few authors as Lombardi have previously explored it [38], but his findings were demonstrated through a case analysis, and could not be taken as evidence to infer universal conclusions owing to its limitations. In our article, we confirm that a positive environmental value will help transform individuals’ desire to protect environment into environmental behavior.

Besides, in this article, we also find that public’s evaluation of environmental governance will inhibit public’s risk perception transforming into environmental behavior. On one hand, for the relationship between risk perception and public’s evaluation of environmental governance, our results indicated that individuals with a higher risk perception are dissatisfied with the performance of governments’ environmental management. One important reason might be that government in China is the main body of environmental governance, the public, acting as the evaluator, would give a high satisfaction level to government, when government officials perform well in environmental management. On the other hand, public’s evaluation of environmental governance is also a crucial factor to predict individuals’ responses to environmental risk in China. It means that individuals with a high satisfaction level with the government’s environmental management will tend to remain silent, that is, not take any action in response to environmental risks.

It should be noted that environmental knowledge is a key factor that act as the bridge between information channel factors (e.g., media use, interpersonal networks) and cognitive evaluation factors (e.g., environmental values, public’s evaluation of environmental governance), which was first examined in this study. Specifically, in this article, we found that both media use and interpersonal networks, on the one hand, have a significant effect on environmental knowledge. This means that information channels can influence individuals’ knowledge level about environmental risk. For example, Adams and Gynnild demonstrated that information channels can significantly enhance the public’s understanding of environmental risk [60]. On the other hand, individuals with greater environmental knowledge would have a lower level of satisfaction with governments’ environmental management, while having a stronger desire to protect the environment. This means that individuals often evaluate the government’s environmental management performance on the basis of their existing environmental knowledge [61].

### 5.2. Evolution Path from Environmental Risk Perception to Behavioral Responses 

By comparing the influence path of radical environmental action, environmental concern action and environmental protection action, this article also demonstrated the evolution paths of the three environmental behaviors under the same level of risk perception, that is, risk transformation and diffusion, risk perception enhancement and risk attenuation, respectively, which will be discussed in this section.

#### 5.2.1. Risk Transformation and Diffusion Path: From Environmental Risk Perception to Radical Environmental Behavior

**Figure 3:**
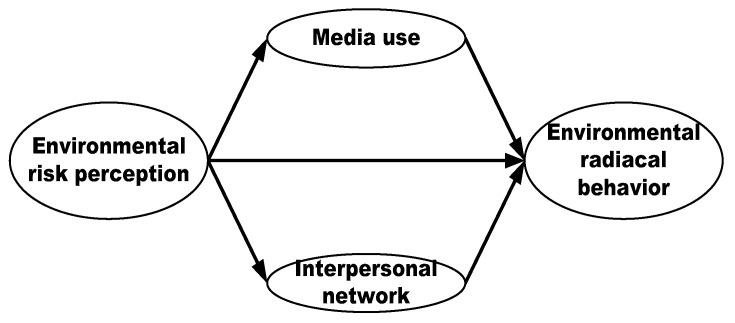
Risk transformation and diffusion path: from environmental risk perception to environmental radical behavior.

Through multivariate path analysis of environmental radical behavior, we can conclude that there are three main influence paths from risk perception to radical environmental behavior (see Figure 3), namely, the direct path from risk perception to radical environmental behavior, and the indirect paths from risk perception, via media use, to radical environmental action, and from risk perception, via interpersonal networks, to radical environmental action. Obviously, in this case, information channel factors (such as media use and interpersonal networks) have an effect of risk transformation and diffusion in the progress from risk perception to radical environmental behavior, that is, both individuals’ environmental risk perception and behavioral responses are influenced by media use or interpersonal networks, while both environmental value and the public’s evaluation of environmental governance fail to influence public’s risk perception transformation into radical environmental behavior. One important reason for this might be that, in the information exploding society, individuals’ risk perception to a certain environmental issue is easily transformed into non-rational action under the action of media or interpersonal networks. For example, Kasperson et al. have indicated that social media act as the “amplification station” of the progress from risk perception to risk behavior [36], which strengthens individuals’ fear and worry about environmental risk, and then may caused street protest events. Therefore, we call the evolution path from environmental risk perception to radical environmental action a “risk transformation and diffusion path”, which was well reflected in the Maoming PX event. In this case, even though Maoming municipal government took various measures, such as information disclosure, risk communication and public participation, etc., to remove the fear about the PX project from the public, thousands of citizens still filled in street to protest the construction of the *para*-xylene (PX) chemical plant in Maoming due to the fast communication of risk information, and even rumors. For example, some rumors like “street protest killed 15 citizens and injured over 300”, “highly poisonous PX” were widely spread among citizens. As a consequence, a large number of citizens from Shenzhen, Guangzhou city also begun to attend the protest. Thus, we can deduce that information channel factors are the main factors that lead to the derivative of environmental radical behavior. 

#### 5.2.2. Risk Perception Enhancement Path: From Environmental Risk Perception to Environmental Concern Behavior

**Figure 4:**
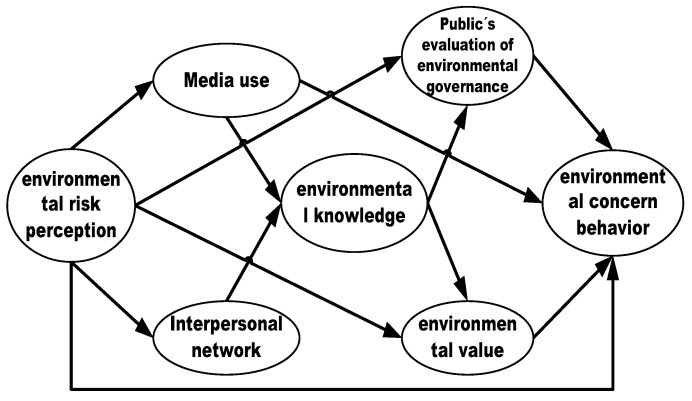
Risk perception enhancement path: from environmental risk perception to environmental concern behavior.

Similarly, we also find that there are eight influence paths from environmental risk perception to environmental concern behavior (see Figure 4), that is, the direct path from risk perception to environmental concern behavior, and the indirect paths that mainly include the path that from risk perception, via media use, to environmental concern behavior; the path from risk perception, via cognitive evaluation factors (such as environmental values and the public’s evaluation of environmental governance), to environmental concern behavior; the path from risk perception, via the information channel factors (such as media use and interpersonal networks), environmental knowledge, the cognitive evaluation factors, to environmental concern behavior. Obviously, in this case, the cognitive evaluation factors (such as environmental value and public’s evaluation of environmental governance) have an effect of risk perception enhancement in the progress from risk perception to environmental concern behavior because all evolution paths but two indicated that cognitive evaluation factors have a significantly mediated effect in the progress from environmental risk perception to environmental concern behavior. For example, although interpersonal networks fail to directly influence individuals’ environmental concern behavior, they plays an indirect effect on environmental concern action by other factors, such as environmental knowledge, and cognitive evaluation factors. Therefore, we can call the evolution path from environmental risk perception to environmental concern behavior a “risk perception enhancement path”. Specifically, individuals with a high level of environmental risk perception will increase their willingness to improve the environment, and their dissatisfaction with governments’ environmental management will increase, thereby leading to environmental concern behavior. This finding about risk perception enhancement paths is similar to those from most existing studies. For example, Douglas and Lash indicated that the public’s environmental values, risk cognitive levels, and their environment, to some extent, increased their perception level of environmental risk [62]. In fact, the “risk perception enhancement path” is well reflected in nature disasters, such as earthquakes, typhoons and floods, etc. In such cases, although individuals often have strong risk perception if a natural disaster occurs, most of them tend to be actively concerned about such environmental risk information, rather than take environmental protection action to respond to such environmental risks. One important reason might be that, even though individuals’ risk perception may be strengthened by information channel factors, the government’s proactive response can alleviate their fear and worry about nature disasters to some extent, and the improvement of satisfaction with the government’s environmental management would push the public to actively pay attention to the disaster risk, rather than take unrational actions. 

#### 5.2.3. Risk Attenuation Path: From Environmental Risk Perception to Environmental Protection Behavior

**Figure 5:**
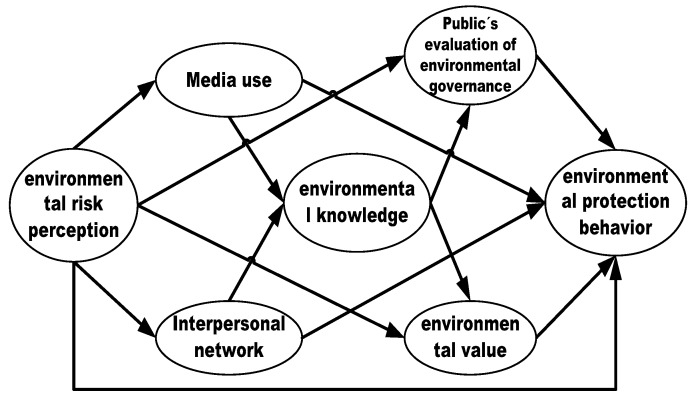
Risk attenuation path: from environmental risk perception to environmental protection behavior.

For environmental protection behavior, we also find that there are nine influence paths from environmental risk perception to environmental protection behavior (see Figure 5). Of these, the direct path is the progress from risk perception to environmental protection action, and the indirect paths mainly include the path from risk perception, via the information channel factors (such as media use and interpersonal networks), to environmental protection action; the path from risk perception, via the cognitive evaluation factors (such as environmental values and the public’s evaluation of environmental governance), to environmental protection behavior; the path from risk perception, via the information channel factors, environmental knowledge and the cognitive evaluation factors, to environmental protection action. Obviously, in this case, both information channel factors and cognitive evaluation factors have an effect of risk attenuation in the progress from environmental risk perception to environmental protection behavior. For example, individuals with high level of environmental risk perception would decrease their social contacts and their environmental knowledge level, which in return attenuates their risk perception. Then, this attenuated risk perception will be transformed into environmental protection action under the action of cognitive evaluation factors. Thus, the influence path from risk perception to environmental protection behavior can be characterized as a “risk attenuation path”. In general, both government’s “desalination treatment” and public’s “powerlessness” towards environmental risks may promote individuals to ignore the potential risk [63], thus they often adopt some self-protection actions to respond to environmental risks. For example, Feng et al. indicated that individuals are willing to pay more to purchase better quality products to reduce or even avoid an associated risk, when facing product quality risks [29]. Besides, Prati et al. also adopt similar views, and they indicated that individuals with higher levels of risk perceptions of pandemic influenza H1N1 are more likely to adopt health-related recommendations to avoid the risk [15]. In fact, the “risk attenuation path” was well reflected in haze pollution incidents. In such cases, although individuals have a high level of risk perception to haze, they cannot do nothing to reduce haze because haze process control is a complicated task that needs considerable resources. Meanwhile, at present, the performance of the government’s haze governance is still not satisfactory. In this case, those individuals with high risk perception level of air pollution often adopt preventive behaviors, such as wearing a mask, minimizing outdoor activities, etc, to cope with the potential damage that haze causes. It is shown that over 90% of citizens often take corresponding action, such as rarely opening windows, growing some flowers indoors, buying low-energy appliances, smoking less, etc., to prevent haze [64].

## 6. Conclusions

In this article, we investigate the influence path of environmental risk perception on behavioral responses in Chinese context from a micro perspective. On the basis of the previous literature, we developed a conceptual model to explore how the public’s environmental risk perception is transformed into different behavioral responses when facing environmental risks in China. To achieve this goal, we adopted the multivariate path analysis method to test the proposed conceptual model, using data from the Chinese General Social Survey 2013 (CGSS2013). The research findings indicated that both information channel factors and cognitive evaluation factors can significantly influence the progress from environmental risk perception to behavioral responses. Of these, both media use and environmental values play an amplification role in the transformation process, while interpersonal networks and the public’s evaluation of environmental governance would inhibit the public’s risk perception transforming into environmental behavior. Besides, environmental knowledge displays a key bridging role between information channel factors and cognitive evaluation factors. These research findings also demonstrated the evolution paths of three environmental behaviors under the same level of risk perception, namely, risk transformation and diffusion, risk perception enhancement and risk attenuation, respectively. 

The main contribution of this article is enhancing the understanding of the progress that how how the public’s environmental risk perception is transformed into different behavioral responses when facing environmental risks in China. Firstly, this study empirically examined which factors influence the progress from environmental risk perception to behavioral responses in China by using the structural equation modeling procedure, unlike the previous literature focusing on case and text analyses. Secondly, we also integrated the environmental behavior strategies on the basis of the research that Lindell & Perry proposed, and constructed a new hypothetical model that aims to understand the evolution path of different environmental behaviors [8]. However, there are some limitations in this study due to the preset research objectives and limited time. For example, our research findings are still limited by the use of cross-section data due to a lack of continuous environmental risk survey data. In addition, it should be noted that exogenous demographic variables, in essence, have a significant structural effect on the process from environmental risk perception to behavioral responses, and previous literatures have proved it [65], and future studies should consider these matters.

## Figures and Tables

**Table 1:** Descriptive Statistics and Inter-correlations of the Constructs.

	M	SD	1	2	3	4	5	6	7	8	9
ERP	26.84	8.87									
MU	10.66	4.18	0.268 ***								
IN	8.12	2.17	−0.087 ***	0.027 *							
EV	0.32	0.76	0.073 ***	0.092 ***	0.010						
EEG	6.15	2.07	−0.131 ***	−0.131 ***	0.010	−0.044 ***					
EK	5.71	2.61	0.199 ***	0.375 ***	−0.027 *	0.093 ***	−0.154 **				
ERB	1.13	0.39	0.118 ***	0.161 ***	0.048 ***	0.035 **	−0.033 **	0.057 ***			
ECB	3.52	1.13	0.165 ***	0.336 ***	0.023 *	0.125 ***	−0.004	0.288 ***	0.199 ***		
EPB	11.35	2.46	0.199 ***	0.328 ***	0.047 ***	0.070 ***	−0.013 *	0.232 ***	0.367 ***	0.199 ***	

Notes: * *p* < 0.05;** *p* < 0.01;*** *p* < 0.001

**Table 2:** Test statistics for hypothesized model.

Model 1	CMIN	df	NFI	CFI	RMSEA	$\chi^{2}$
Fitting index	112.424	8	0.954	0.957	0.046	14.053
Model 2	CMIN	df	NFI	CFI	RMSEA	$\chi^{2}$
Fitting index	320.608	8	0.902	0.904	0.080	40.076
Model 3	CMIN	df	NFI	CFI	RMSEA	$\chi^{2}$
Fitting index	198.955	8	0.937	0.939	0.062	24.869
